# Investigating the conditions in which women GPs thrive: a realist review protocol

**DOI:** 10.3399/BJGPO.2024.0275

**Published:** 2025-07-16

**Authors:** Ruth Abrams, Laura Jefferson, Su Golder, Lilith Whiley, Sophie Park, Vickie Williams, Ruth Riley

**Affiliations:** 1 School of Health Sciences, University of Surrey, Guildford, UK; 2 Department of Health Sciences, University of York, York, UK; 3 University of Sussex Business School, Brighton, UK; 4 Nuffield Department of Primary Care Health Sciences, University of Oxford, Oxford, UK

**Keywords:** women, general practitioners, primary health care, thriving, realist

## Abstract

**Background:**

Women now make up approximately half of the GP workforce. However, many are leaving the profession. This could be because they experience higher rates of burnout, stress and anxiety, suicide, and lower rates of career progression than men. They also take on a greater load of emotional labour compared with men. Retaining this staff group is one of five priorities for future policy and research.

**Aim:**

To synthesise the available evidence on how general practice workplaces can best support women GPs to thrive at work.

**Design & setting:**

We propose to undertake a realist review, which seeks to understand why an approach may work in specific contexts. This involves building an understanding of how contextual factors affect the activation of mechanisms (that is, changes in participant reasoning or behaviours) to produce their outcomes.

**Method:**

We will locate available evidence on the topic, and, using a realist logic of analysis, develop an understanding as to how, why, for whom, and in what contexts women GPs thrive at work. Evidence will include academic literature, policy documents, media items, and guidelines.

**Results:**

Findings will be co-disseminated with public and patient involvement (PPI) and stakeholder members to all key groups, including policymakers, employers, the public, and academic audiences, by a wide variety of means.

**Conclusion:**

This review is intended to improve understanding of how working environments affect women GPs. It is anticipated that findings will support the implementation of strategies to better support this group to thrive at work.

## How this fits in

Women GPs make up half of the workforce yet they are disproportionately affected by burnout, stress, lower rates of career progression, and higher rates of intention to leave than men. The Royal College of General Practitioners contends that retaining this staff group is one of five priorities for future policy and research; therefore, we need to better understand ways to support this workforce. One possibility is offered through the positive psychological notion of ‘thriving at work’, which can enhance both individual health outcomes (such as lowering burnout) and organisational level outcomes (such as commitment, job satisfaction, and retention). This realist review will identify and investigate the conditions in which women GPs can thrive in general practice.

## Introduction

Women GPs are disproportionately affected by higher rates of burnout, stress, anxiety, suicide, and lower rates of career progression.^1–3^ Women doctors also take on a greater load of emotional labour than men, demonstrating higher levels of empathy and spending longer talking to patients about their emotions and feelings.^4,5^ Although patients value being seen by women GPs, this additional investment in patient care can contribute to higher rates of burnout and subsequent intention to leave.^6^ Indeed, large numbers are leaving the workforce.^7^ Women GPs also experience one of the worst pay gaps in medicine.^8^ Of those who do not leave, increasing numbers are lowering their working or clinical hours to mitigate against stress and provide greater work–life balance. This decision appears to affect women more than men, influencing women GPs’ careers.^1,3,9^ This presents a stark backdrop, and is particularly significant given women currently make up 53% of the GP workforce (based on fully qualified full-time equivalents).^10^

This existing evidence naturally has implications for women GPs, employers, and patient care, including negative impacts on individual wellbeing, organisational cost, GP retention, and care quality and safety.^11^ Further evidence is required to better understand how women GPs can be supported to thrive in the workplace and mitigate the effects of gendered discrimination. Thriving at work is the state of functioning positively in mental, physical, and social domains.^12^ It is a multi-faceted state that is associated with several individual and organisational outcomes such as enhanced health and reduced burnout and organisational commitment.^13,14^ Thriving at work is known to facilitate a healthy workplace and sustainable performance.^15^

The ability to thrive (or not) is context specific; what helps some to thrive, may not be the same for others. Given these potential benefits of thriving at work for women GPs, patients, and organisations, an evidence synthesis, and specifically a realist review, is crucial to facilitate understanding that may support health service providers to support women GPs. Realist reviews take into account socio-cultural, socioeconomic, ethical, legal, political, geographical, and epidemiological factors, alongside implementation theory, process, agents, strategies, and outcomes. Taking a realist approach specifically accommodates the transferability of findings across settings. This study aims to identify and investigate the conditions in which women GPs thrive at work; and produce evidence-based recommendations for women GPs, managers, employers, training providers, and policymakers to more effectively support and retain this staff group.

### Research questions

1. What are the mechanisms acting at an individual, team, organisational, and societal level that affect women GPs’ health and wellbeing; performance; and career progression?
2. In what contexts are these mechanisms triggered (or not triggered)?
3. What outcomes (both intended and unintended) do these contexts and mechanisms lead to?
4. What interventions or strategies can be implemented to help women GPs to thrive at work, based on relevant contexts, mechanisms, and outcomes?
5. What are the critical gaps in the literature (including the views and experiences of women and minoritised women)?

## Method

### Study design

**Figure 1.**
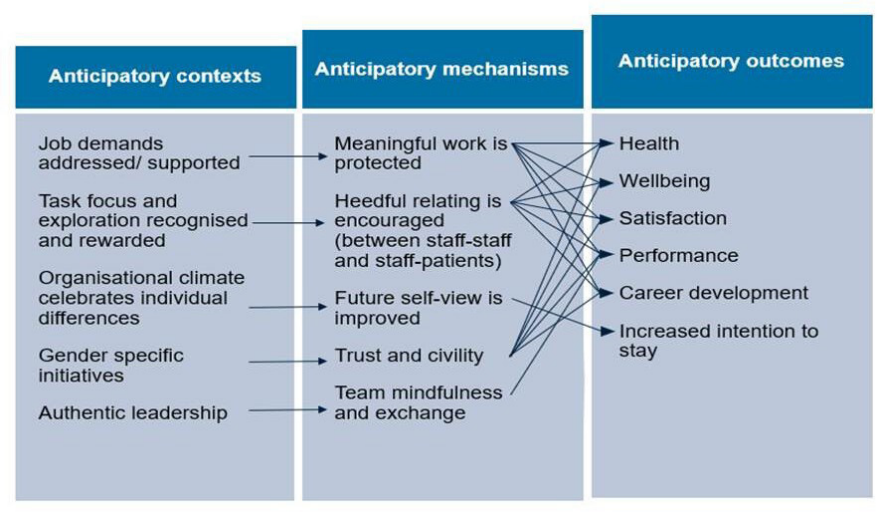
Conceptual framework^17^

We will undertake a realist review and in doing so map approaches to thriving at work, to produce evidence-based recommendations for what could be implemented to support and retain women GPs. Realist reviews are theory-driven and synthesise literature about complex social phenomena including interventions, strategies, and approaches. They focus on understanding the mechanisms by which interventions or strategies work (or not) and seek to understand contextual influences on whether, why, how, and for whom these might work.^16^ Our broad conceptualisation of the relationship between work and thriving is described elsewhere,^17^ and is shown in Figure 1. This is not a gender-specific model; however, we will be coding for intersectionality across our data. We will use this model a priori to (a) help guide our search strategy; (b) classify individual worker characteristics, workplace policies, or workplace conditions; and (c) as a starting point to identify and describe potential mechanisms.

### Research plan

The plan of investigation will follow a detailed realist review protocol informed by Pawson *et al’*s five iterative stages,^16^ and both the RAMESES realist review publication standards, and quality and reporting standards.^18,19^ The review is registered with PROSPERO, which helps avoid duplication and enhance transparency. An initial programme theory (that is, a complex mapping of how an intervention is assumed to work) will be developed at the outset of the project, generated from literature identified in our original research proposal, through preliminary conversations with our stakeholder and PPI groups, and our conceptual model (Figure 1).^17^

The current evidence base on how to support women GPs to thrive at work is disparate. Rather than limit by subject, we will run searches across relevant medical and social sciences databases when we conduct our primary search. In this formal, primary search we will look for studies that discuss women GPs’ health and wellbeing; career development; and/or performance. Searches will be run in academic databases including MEDLINE, Embase, CINAHL Ultimate, PsycINFO, Science Citation Index Expanded (SCIE), Social Science Citation Index (SSCI), and Scopus. A search on Google Scholar will also be undertaken. Bibliographies of retrieved full-text papers will be scanned to identify further references. Forward citation searching will also be undertaken of relevant papers. Search strategies will comprise search terms, synonyms, and index terms for the facets of thriving and women GPs. All searches will be fully reported. We will then engage in follow-up searches, of a more targeted nature in business and management-specific databases. For example, we will conduct a targeted search of empirical evidence on thriving at work, to better understand effective interventions or strategies. See Table 1 for the study inclusion and exclusion criteria.

**Table 1.** Study inclusion and exclusion criteria

Inclusion	Exclusion
Primary data	Secondary data (for example, reviews). However, literature reviews will be saved for citation tracking
All study designs	Book chapters; theses or dissertations; conference proceedings; protocols
Population: women GPs	
OECD countries, particularly those with a similar system to the UK (for example, free at the point of access; GP as gatekeeper)	Low-to-middle income countries; countries with different primary care settings (for example, US)
General practice or primary care (and if necessary, other healthcare settings)	
Studies from 2012 to current	Studies from 2011 and earlier
Any strategies, interventions, or approaches that support women GPs at work including process evaluations; studies exploring experiences; and barriers and facilitators	
Outcomes of interest include: health and wellbeing, OR career development (including job satisfaction or progression) OR performance (including retention)	
Relevant grey literature	

OECD = Organization for Economic Cooperation and Development

**Figure 2.**
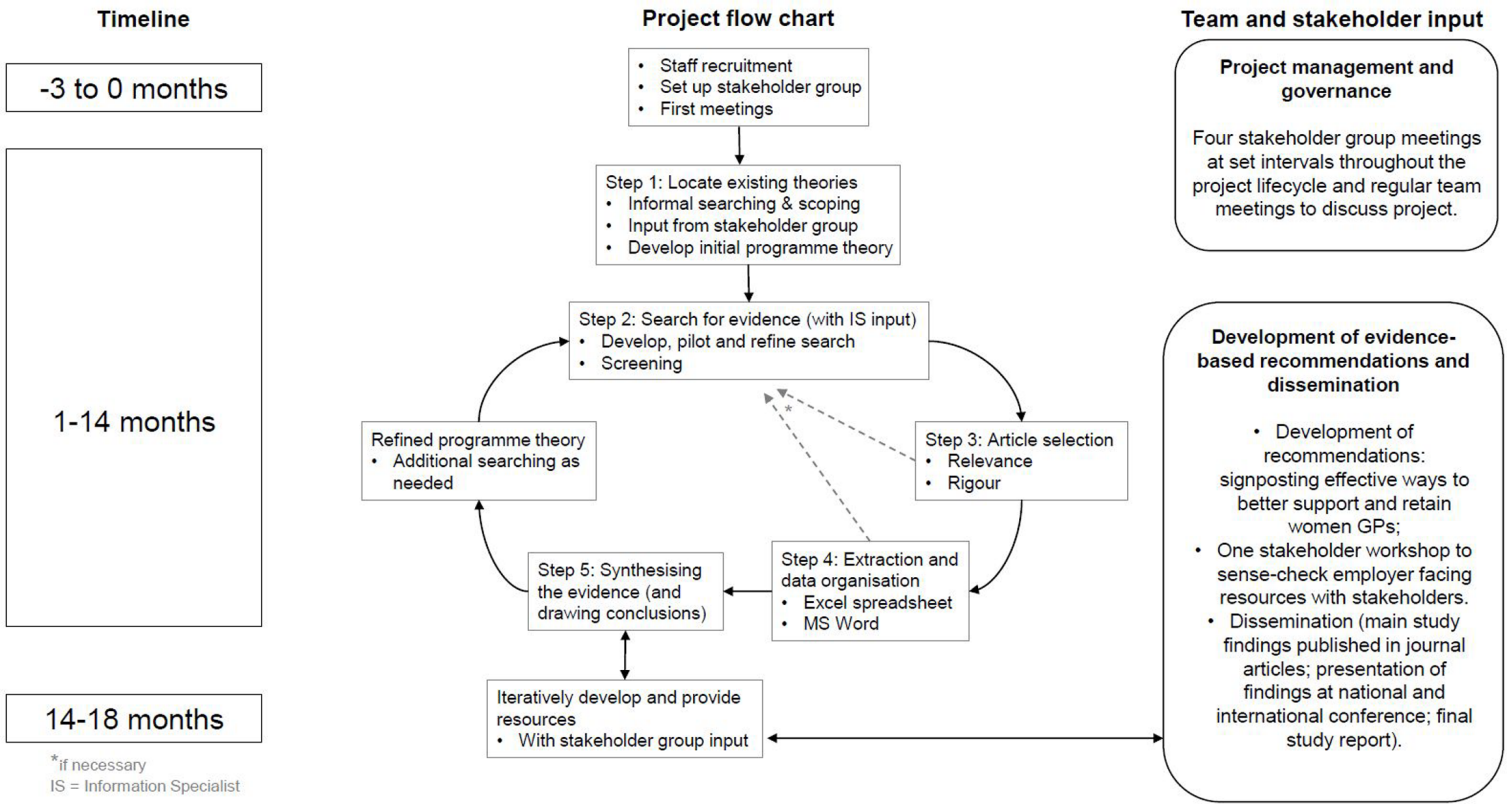
Study flowchart.*If necessary. IS = information specialist

Following a realist logic of analysis (see, for example, Treacy *et al* ^20^), we will map these bodies of literature against each other to identify where the transferability of literature about thriving more generally, maps specifically onto the working conditions of women GPs, based on our understanding and knowledge from our primary search. For further information on the specific study steps, see Figure 2, which is an adapted version of Wong *et al.* ^21^

### Stakeholder and patient involvement

This review will draw on both stakeholder and patient and public involvement (PPI). Our PPI group consists of two practising GPs, and three patients. Our stakeholders include a range of content matter experts across the field of primary care; medical education; organisational psychology; and health and wellbeing at work. Both groups will be invited into the project at specific intervals relevant to project milestones. Meetings are designed to be accessible and inclusive.

## Discussion

### Summary

Immediate policies are necessary to solve the current workforce crisis and in particular how to support women GPs. One possibility is offered through the notion of ‘thriving at work’.^14^ There is a growing body of literature on the concept of thriving at work that explores conditions for both individuals and organisations. However, this concept has not yet translated into the field of general practice, nor to women specifically, and there is an important need to explore the role of context, including intersectionality (that is, race, sexuality, social class, and gender) so as to avoid individual reductionism, and to ensure research that is inclusive and that findings are transferable. Our review of the evidence intends to do just that, therefore aligning to the recent National Institute for Health and Care Research (NIHR) research inclusion strategy.^22^

### Strengths and limitations

This review will be relevant for both women GPs in general practice and potentially women who work in different care settings and caring professions. This indicates multiple access points for change at both the level of how we conceptualise the notion of ‘thriving’ for women GPs and at the practical level (that is, training, in practice) in terms of how to foster it. This proposal may also hold national and international relevance, particularly for countries with a similar primary healthcare workforce, including Australia, New Zealand, and Canada, which also face healthcare workforce retention problems. It is likely that additional searches will need to be undertaken, outside the healthcare sphere, to identify evidence on thriving. This approach is compatible with a realist review.

### Implications for research and practice

By reviewing literature across disciplines, this proposal will identify the conditions in which women GPs can thrive, and in doing so we will produce an integrated and pragmatic set of recommendations. The anticipated implications of these outputs include (a) improved understanding of the range of factors and contexts that affect women GPs’ ability to thrive or not at work; (b) available interventions or strategies capable of being implemented in order to better support and retain women GPs; and (c) future research capable of measuring intervention success. We anticipate that our evidence-based recommendations for policymakers, employers, training providers, and GPs will help to guide decision making about whether interventions are needed; and if so, which are most effective, in what circumstances.

## Data

The dataset relied on in this article is available from the corresponding author on reasonable request.

## Trial registration

CRD42024601806.
